# Identification of suitable endogenous control genes for microRNA gene expression analysis in human breast cancer

**DOI:** 10.1186/1471-2199-9-76

**Published:** 2008-08-21

**Authors:** Pamela A Davoren, Roisin E McNeill, Aoife J Lowery, Michael J Kerin, Nicola Miller

**Affiliations:** 1 Department of Surgery, National University of Ireland, Galway, Ireland

## Abstract

The discovery of microRNAs (miRNAs) added an extra level of intricacy to the already complex system regulating gene expression. These single-stranded RNA molecules, 18–25 nucleotides in length, negatively regulate gene expression through translational inhibition or mRNA cleavage. The discovery that aberrant expression of specific miRNAs contributes to human disease has fueled much interest in profiling the expression of these molecules. Real-time quantitative PCR (RQ-PCR) is a sensitive and reproducible gene expression quantitation technique which is now being used to profile miRNA expression in cells and tissues. To correct for systematic variables such as amount of starting template, RNA quality and enzymatic efficiencies, RQ-PCR data is commonly normalised to an endogenous control (EC) gene, which ideally, is stably-expressed across the test sample set. A universal endogenous control suitable for every tissue type, treatment and disease stage has not been identified and is unlikely to exist, so, to avoid introducing further error in the quantification of expression data it is necessary that candidate ECs be validated in the samples of interest. While ECs have been validated for quantification of mRNA expression in various experimental settings, to date there is no report of the validation of miRNA ECs for expression profiling in breast tissue. In this study, the expression of five miRNA genes (*let-7a, miR-10b, miR-16*, *miR-21 *and *miR-26b*) and three small nucleolar RNA genes (*RNU19, RNU48 *and *Z30*) was examined across malignant, benign and normal breast tissues to determine the most appropriate normalisation strategy. This is the first study to identify reliable ECs for analysis of miRNA by RQ-PCR in human breast tissue.

## Background

Approximately 98% of the human transcriptome is non-protein-coding RNA (ncRNA) [1,2]. The fraction of ncRNA believed to be functional in cells was once limited to the well-characterised transfer and ribosomal RNAs. However, this fraction has recently been expanded to include microRNAs (miRNAs), a class of short, single-stranded RNAs that target, through nucleotide complementarity, specific messenger RNAs, enabling them to negatively modulate gene expression. First characterised in 1993, miRNAs were initially shown to be involved in the control of developmental timing in *Caenhorhabditis elegans *[3]. Now, 15 years later, 541 human miRNAs have been submitted to the most recent edition of the online miRNA sequence repository, miRBase [4,5]. A very small proportion of identified miRNAs have verified roles, in processes such as cell proliferation [6] and apoptosis [7]. It may be some time before the full catalogue of biologically-functional miRNAs is compiled but the involvement of miRNAs in the regulation of cancer-related genes at the post-transcriptional level has already led to miRNAs being hailed as a novel class of tumour suppressor genes and oncogenes and the coining of the term "oncomiR" [reviewed in [8] and [9]]. Further elucidating our limited understanding of the mechanisms of metastasis [10,11]; the spread of cancer cells from the primary neoplasm to distant organs, has become a major focus in miRNA studies [12-14]. Gene signatures from these studies will contribute to our understanding of the multi-step processes of metastasis and may also enable advanced indication of the likelihood of tumour invasion and metastasis based on the characteristics of the primary tumour.

The expression of miRNAs has been studied using traditional, semi-quantitative methods such as northern blotting [15], bead-based flow-cytometry [16] and microarray technology [17]. However, by far the method of choice for expression quantitation is real-time quantitative PCR (RQ-PCR) due to its sensitivity, wide dynamic range and low template requirements. The technique has itself been revolutionised in recent years with the development of stem-loop primers that specifically convert the mature, functional miRNA into its DNA complement [18]. Furthermore, a multiplex stem-loop RQ-PCR format is currently being refined to allow multiple miRNAs to be transcribed simultaneously [19,20].

To produce reliable relative RQ-PCR data, corrections must be made for variation between reactions introduced during the steps from sample preparation to amplification. Incorporating an endogenous control (EC) gene into the experimental design is an effective method of normalising the data but candidate ECs must be tested on a representative number of the sample population if not the entire sample set [21-23]. Use of an unreliable EC may lead to inaccurate, unreliable results and previous studies show that mRNA expression can be made to appear up- or down-regulated by one order of magnitude based solely on the choice of EC [21].

Using the Medical Subject Heading (MeSH) terms microRNAs, neoplasm and reverse transcriptase polymerase chain reaction, a recent PubMed search returned 42 articles, 5 of which detailed miRNA RQ-PCR expression profiling studies using human neoplastic breast samples. The EC(s) used in these studies were *let-7a *and *miR-16 *[24], *U6 *small nuclear RNA (snRNA) and tRNA for initiator methionine [25], *18S rRNA *[26], glyceraldehyde-3-phosphate dehydrogenase (*GAPDH*) [27] and in one article, the EC used for RQ-PCR analysis was not given [28]. There is currently no consensus on suitable ECs for quantitative analysis of miRNA by RQ-PCR in human breast tissue. Concern has been expressed regarding the use of ribosomal RNAs in normalisation strategies as they can be expressed at much greater levels than the target RNA making it very difficult to quantify an rRNA and a rare transcript in the same RNA dilution [22,29,30]. Moreover, there is evidence of rRNA deregulation in apoptosis [31]. It is also clear that the traditionally-used but seldom validated *GAPDH *and β-actin (*ABTB*) genes are not suitable endogenous controls for some studies [32-34]. Our aim was to identify from a panel of RNA species similar to miRNAs, suitable EC candidates for miRNA analysis by RQ-PCR.

The expression of eight small RNAs was determined in 36 fresh-frozen breast tissues; three small nucleolar RNAs (snoRNAs, *RNU19, RNU48 *and *Z30*) and five miRNAs (*let-7a, miR-10b, miR-16, miR-21 *and *miR-26b*). The five miRNAs were chosen as candidate ECs for this study based on their known expression in human breast tissue and/or their previous use as an EC gene for miRNA RQ-PCR analysis [24]. The three snoRNA genes were selected from a panel of ten commercially available TaqMan control assays. SnoRNAs, found within the nucleolus, range from 60–300 nt in length and chemically modify rRNA [35], small nuclear RNA (snRNA) [36] and mRNA [37] through their recognition of short sequences in the target molecule and recruitment of associated proteins to the site. MiR-30*, previously referred to as miR-30a-3p, targets RNA involved in several cancer-related biological processes [38] and was chosen as a target gene to investigate the effect of EC gene selection on relative quantitation. Downregulation of miR-30* has previously been shown to increase transcription of mRNAs involved in processes such as angiogenesis (thrombospondin I, cysteine-rich, angiogenic inducer) and cell cycle transition (cyclin-dependent kinase 6) [38]. Samples consisted of malignant (n = 21), benign (n = 5) and normal (n = 5) breast tissues. Malignant tumour tissues were representative of all tumour grades and hormone receptor states. The malignant breast tumour tissues were divided into three groups depending on the patient's disease progression in the five years following removal of the primary tumour; tumours from patients who did not develop metastases (metastases-free, MF, n = 13), those from patients who developed bone metastases (BM, n = 7) and those from patients who developed visceral and bone metastases (VBM, n = 6).

## Results

### RNA analysis

Concentrations of small RNA ranged from 45–431 ng μL^-1^. The distribution of miRNA yields (RNA in the 10–35 nt range) across the various malignant groups, benign and normal tissues was determined using the Agilent Small RNA Assay (Table 1). Percentage of miRNA in the small RNA fraction ranged from 12%–98%. For the majority of samples, miRNA comprised 26–75% of total small RNA. High miRNA yields (> 75% miRNA) were seen in samples from the BM and MF groups. Low yields (< 25% miRNA) were seen in all groups apart from the normal tissue group. The large RNA samples, extracted separately but at the same time as the small RNA used for this study, all had an RNA integrity number (RIN) ≥ 7.

**Table 1:** miRNA yield from small RNA-enriched fractions according to tissue group

Tissue group\Percentage of miRNA in small RNA-enriched fraction	**1–25%**	**26–50%**	**51–75%**	**76–100%**
**MF**	3	4	3	3
**BM**	1	1	2	3
**VBM**	1	5	0	0
**BEN**	1	2	2	0
**Normal**	0	1	4	0

***Total***	6	13	11	6

Extracted RNA, enriched for small RNA, was analysed using the Agilent Small RNA Assay. The percentage of miRNA (10–35 nt) in each small RNA-enriched sample (< 200 nt) was determined.

**Abbreviations: **MF = metastases free, BM = bone metastases, VBM = visceral and bone metstases, BEN = benign.

### Relative Expression Quantitation

**Figure 1:**
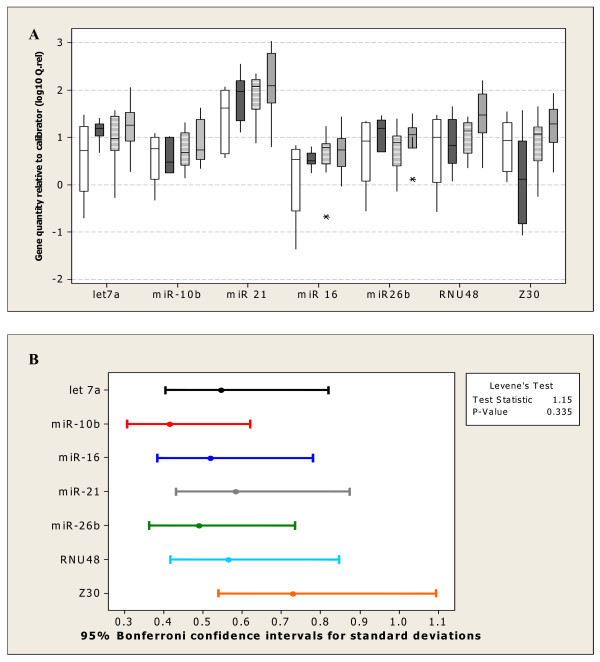
**Relative quantity of each candidate EC**. **(A) **Quantity of the candidate endogenous control genes let-7a, miR-10b, miR-21, miR-16, miR-26b, RNU48 and Z30, relative to calibrator (normal tissues) and corrected for amplification efficiency (Q. rel = E^-ΔCt^), in the benign (BEN, clear ), bone metastases (BM, dark), metastases free (MF, dashed) and visceral and bone metastases (VBM, shaded) groups. The boxes show the interquartile range and median, whiskers indicate the range and outliers are depicted with the symbol (*). No difference was found within gene between the tissue subgroups (P>0.05) thus establishing the validity of EC comparison. **(B) **Variation associated with candidate endogenous control genes. Relative quantity of each gene is relative to calibrator (normal tissues) and corrected for amplification efficiency (Q.rel = E^-ΔCt^). There was no significant difference in variance associated with relative gene expression (P > 0.05).

The threshold cycle (C_t_) is the amplification cycle number at which the fluorescence generated within a reaction rises above a defined threshold fluorescence [39]. The eight candidate ECs displayed a wide expression range with C_t _values between 18 and 36. MiR-16 and miR-21 showed relatively high expression with median Cts of 21, while let-7a, miR-10b, miR-26b and RNU48 were moderately abundant with median Cts of between 23 and 27. Z30 had lower abundance with a median C_t _of 29. RNU19 expression was very low in these samples with Cts ranging from 26.2 to 38.9. It was decided to exclude RNU19 from further analysis due to its low expression. Ct values were converted to relative quantities (Q.Rel) using the formula: E^-ΔCt^, where E = PCR amplification efficiency and ΔCt = average Ct, test sample-average Ct, calibrator sample. There was no significant difference in variance between genes (P > 0.05, Fig. 1B). The relative quantities did not differ significantly between the MF, BM, VBM and BEN groups for any of the candidate ECs (P > 0.05; Fig. 1A). As NormFinder and geNorm assume candidates are not differentially expressed between groups, this analysis is necessary to validate use of these methodologies [40]. Equivalent expression between the tumour tissues (benign and malignant) and the normal breast tissues (used as the calibrator) was confirmed for each of the seven candidate ECs using the equivalence test and a fold change cut-off of 3[41]. All genes, with the exception of Z30, were also equivalently expressed between the malignant and benign tumour groups using a fold change cut-off of 3.

### Stability Candidate EC Expression

Stability of the candidate ECs was examined using geNorm and NormFinder. The ranking of the candidates, as determined by these programmes, is shown in Table 2. The lower the stability value, the higher the gene stability. With a stability value of 0.312, NormFinder selected let-7a as the most stably expressed single gene. The best combination of two genes, let-7a and RNU48, further reduced the NormFinder stability value to 0.221. Two out of the seven genes showed geNorm stability measures (M) below the default limit of 1.5 and the programme identified let-7a and miR-16 as the most stable pair of ECs (M = 0.978).

**Table 2:** Ranking of candidate EC gene and choice of best pair of EC genes by NormFinder and geNorm programmes

**Rank**	**NormFinder^a^**		**geNorm^b^**	
	
	**Gene**	**Stability**	**Gene**	**Stability (M)**
1	let-7a	0.312	let-7a	1.427
2	miR-16	0.379	miR-16	1.473
3	RNU48	0.401	miR-26b	1.538
4	miR-26b	0.425	RNU48	1.567
5	miR-10b	0.435	miR-10b	1.667
6	miR-21	0.601	miR-21	1.692
7	Z30	0.624	Z30	2.272
Best combination	let-7a/RNU48	0.221	let-7a/miR-16	0.978

Greater expression stability is indicated by a lower stability value (M). Results for seven EC candidates are given as RNU19 was excluded from analysis due to its low expression in the breast tissues. For NormFinder analysis, breast tissue samples were grouped into metastases-free (MF), bone metastases (BM), visceral and bone metastases (VBM) and benign (BEN). The stability is calculated from the intra- and inter-group variation and the best combination of EC genes is also given. ^b ^GeNorm stability is based on an estimate of the pairwise variation (M).

### Effect of EC on Relative Quantity of miR-30*

**Figure 2:**
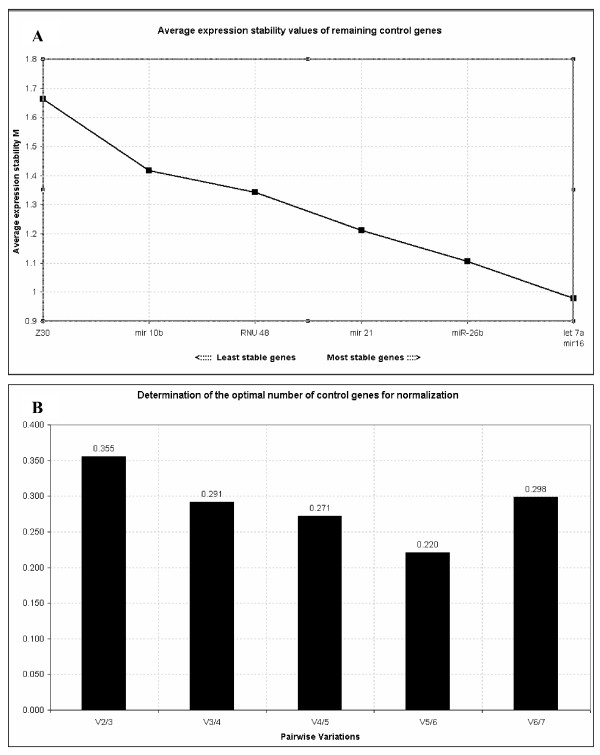
**GeNorm analysis of candidate ECs**. **(A) **Average expression stability of the EC candidates as calculated using GeNorm. A stability value (M) was calculated for each candidate EC. The least stable gene with the highest M value was automatically excluded and M values recalculated for the remaining ECs, ultimately resulting in a stability value for the two most stable ECs. **(B) **Determination of the optimal number of ECs for normalisation. The V value defines the pairwise variation between two sequential normalisation factors. GeNorm indicated optimal normalisation of gene expression could be achieved using the top five most stable ECs.

To assess the effect of EC on relative quantitation of the target gene, miR-30*, this miRNA was normalised using each of the candidate EC genes in turn. Depending on the normaliser, miR-30* expression was either significantly different between tissue groups (P < 0.05) or no differences were detected. When normalised using miR-26b, ranked in the top four mosts Table 3 andidates by both geNorm and NormFinder (Table 2), no differences were detected between tissue groups. Normalisation to all other individual ECs detected significant differences between the BM and VBM tissue groups. Only normalisation with RNU48 detected a significant difference in miR-30* expression between the MF and VBM tissue groups. GeNorm selected let-7a and miR-16 as the most stable EC pair and let-7a was selected as the single most stable EC gene using NormFinder. Thus the effect of using either let-7a as a single gene or using the recommended EC pair, let-7a and miR-16, on miR-30* expression was assessed. Significant differences in miR-30* expression were detected between tissue groups using either the one EC (P = 0.007) or the two EC (P = 0.01) approach, however the BM and MF tissue groups were found to be significantly different using the EC pair, let-7a and miR-16 but this was not detected when let-7a was used as the sole EC gene. The lowest pairwise variation value (V) was generated using the top five genes from the panel, indicating that this would be the most stable EC gene set to use. MiR-30* was normalised using the top two EC genes and using the top five EC genes to assess what effect this would have on miR-30* relative quantification. Significant differences in miR-30* expression were detected between the tissue groups using either the top two ECs (P = 0.01) or the top five ECs (P = 0.002, Fig. 2B), however the *post-hoc *analyses varied slightly in that the two gene normalisation detected a difference between the BEN and MF tissue groups not detected by the five EC gene approach. Conversely, the 5 gene approach identified a significant difference in miR-30* expression between the MF and VBM tissues, not detected when using the most stable pair. Both normalisation strategies did detect significant differences between the BM vs MF groups, the BM vs VBM groups and the BEN vs VBM groups.

**Table 3:** Clinical and pathological data on malignant tumour samples where available

*Patient* *Number*	*Patient* *Age*	*Menopausal* *status*	*Size* *(mm)*	*T*	*N*	*M*	*Grade*	*ER*	*PR*	*HER2/* *neu*	*Subtype*	*Metastatic* *grouping*
1	41	Pre	25	2	0	0		P			Luminal A/B	MF
2	50	Pre	35	2	1	0	1	P	P	N	Luminal A	MF
3	38	Post	35	2	1	0	1	P	P	N	Luminal A	MF
4	43	Pre	10	1	0	0	1	P	P	N	Luminal A	MF
5	49	Post	20	1	1	0	2	P	N	N	Luminal A	MF
6	78	Post	20	1	1	0	1	P	P	N	Luminal A	MF
												
7	51	Post	18	1	0	0		N		N	LuminalA/Basal	MF
8	75	Post	36	4	1	0	2	P			Luminal A/B	MF
9	59	Post	50	2	1	0	3	P	P	N	Luminal A	BM
10	53	Post	85	3	1	0	3	N	N	N	Basal	MF
11	43	Pre	50	2	1	0	3	P	N	N	Luminal A	MF
12	69	Post	35	4	2	0	3	P	P	N	Luminal A	VBM
13	66	Post	12	1	0	0	3	P	N	N	Luminal A	MF
14	58	Post	20	4	1	0	2	P	P	N	Luminal A	MF
15	58	Post	15	1	1	0	2	P	P	N	Luminal A	VBM
16	70	Post	20	1	0	0	2	P	P	N	Luminal A	MF
17	52	Post	25	4	1	0	3	P	P	P	Luminal B	VBM
18	78	Post		1	0	0		P	P	N	Luminal A	BM
19	61	Post	33	2	1	0	1	P	P	P	Luminal B	BM
20	48	Pre	30	2	1	0	3	P	P	N	Luminal A	VBM
21	50	Pre	30	2	1	0	3	N	N		Basal/HER-2	VBM
												
22	51	Post	20	1		0	1	P			Luminal A/B	VBM
23	69	Post	40	2	1	0	2	P	N	N	Luminal A	BM
24	58	Post	21	4		0	3	N	N	P	HER-2	BM
25	61	Post	35	2	1	0	3	P	P	N	Luminal A	BM
26	64	Post	15	1	1	0	2	P	N	P	Luminal B	BM

T, N and M refer to the primary tumor size, nodal status and distant metastases status according to the TNM breast cancer classification system. ER: = oestrogen receptor status; PR: = progesterone receptor status and HER2/*neu *= v-erb-b2 erythroblastic leukaemia viral oncogene status. Where data was not available for ER, PR and HER-2, possible subtype based on available hormone receptor status is given. Metastatic groupings refer to patient status five years after presentation; patients were either metastases-free (MF), had developed bone metastases (BM) or had developed visceral and bone metastases (VBM).

## Discussion

In recent years, miRNAs have emerged as key players in tightly-controlled biological processes such as proliferation [6], apoptosis [7,42] and tumour invasion [13]. MiRNAs, first implicated in malignancy in 2002 [43], are known to be deregulated and/or mutated in numerous cancers including breast cancer [44] and there is evidence to suggest that miRNA expression profiles may be more accurate in classifying breast cancer subtypes than mRNA expression profiles [16].

Adaptation of traditional RNA isolation and reverse transcription protocols has facilitated the application of RQ-PCR to the study of miRNA expression. Mature miRNAs were amplified and quantified using PCR for the first time in 2005 [18,45] and recent developments include a 220-plex RQ-PCR allowing the analysis of multiple miRNAs from single cells [19]. The high sensitivity of RQ-PCR demands appropriate normalisation to correct for non-biological variation and the use of EC genes remains the most commonly used method. The issue of carefully selecting and validating EC genes has already been discussed for a number of experimental systems in the context of RQ-PCR for mRNA [21,29,46] however, this issue has not yet been addressed in relation to the relative quantitation of miRNA in breast tissue. This issue is particularly pertinent to the area of miRNA studies utilising RQ-PCR since it is still common practice to synthesise a gene-specific cDNA for each sample, using miRNA-specific primers, thereby introducing additional non-biological variation not accrued during the synthesis of cDNA from mRNA when using random or oligo-dT primers.

This paper describes the first systematic assessment of candidate ECs for the normalisation of miRNA RQ-PCR data in breast cancer. In the rapidly evolving field of miRNA reasearch, consensus has not yet been reached on how best to tackle this issue. Numerous RNA species, including rRNAs, tRNAs, snRNAs and miRNAs have previously been used as ECs in miRNA RQ-PCR studies of the breast. Concern has been expressed regarding the use of rRNAs in normalisation strategies as they can be expressed at much greater levels than the target RNA making it very difficult to quantify a rRNA and a rare transcript in the same RNA dilution [22,29,30]. Moreover, a role for rRNA in apoptosis [31] and cancer [47] has been reported. A proportion of snRNAs and snoRNAs may exhibit tissue-specific and developmental regulation [48] emphasising the need for validation of commercially-available control assays. U6 snRNA (RNU6B), commonly used to normalise miRNA RQ-PCR data [49,50] was found to be less stably expressed than let-7a and miR-16, the EC pair proposed by this study [51].

This is the first report detailing the percentage of miRNA retrieved in small RNA-enriched fractions of primary breast tissue. RNAs detected using the Agilent small RNA assay include miRNAs, smaller ribosomal RNAs such as the 5.85S (154 nt) and 5S (117 nt) subunits, transfer RNAs (73–93 nt) and snoRNAs (60–300 nt). Perhaps unsurprisingly, we found the proportion of miRNA in the small RNA sample ranged from 12–98%. The varying miRNA yields were well distributed amongst the tissue groups. The variation in ratio was not dependent on the type of tissue, on the RNA extraction or on the total yield of the RNA. In this laboratory we also found a much lower proportion of miRNA, ranging from 1–5%, in small RNA extracted from commonly used breast cancer cell lines such as MCF-7, SK-BR-3, T47D and ZR-75-1 (data not shown). A suitable EC gene will have to reflect such changes in the global miRNA population. The variation in miRNA yields and ratios may reflect genomic alterations, common in cancer (reviewed in [52]. It has been shown previously that miRNA frequently map to such regions of instability [53]. This finding raises concerns over how much of the small RNA used for cDNA reactions and other applications is actually the RNA species of interest, and this is especially relevant in studies employing non-miRNA ECs.

Normfinder and geNorm were used to identify suitable ECs for the relative quantitation of miRNA in fresh-frozen primary breast tissue. There was no effect of tissue group on scaled EC expression (P > 0.05, Fig. 1). As previously stated [21] this is an important validation prior to use of geNorm and NormFinder as these models assume candidates are not differentially expressed between experimental groups. The absence of a significant difference in EC expression between groups does not necessarily equate to equivalent expression. Equivalence of expression was assessed using the equivalence test [41]. Equivalent expression between the independent tumour and normal breast tissues was confirmed for all ECs using a fold change cut-off of 3. Equivalent expression between the malignant and benign tumour groups was also assessed and was confirmed for all ECs with the exception of Z30 using the same cut-off. Using the benign, MF, BM and VBM subgroups, NormFinder calculated the intra- and intergroup variations and identified let-7a as the single most stable EC with a stability value of 0.312. However, the use of more than one EC is believed to increase the accuracy of quantitation compared to the use of a single EC [30,54,55] and use of let-7a alone would therefore not be recommended. The EC gene pair, let-7a and RNU48 had an improved NormFinder stability value of 0.221.

**Figure 3:**
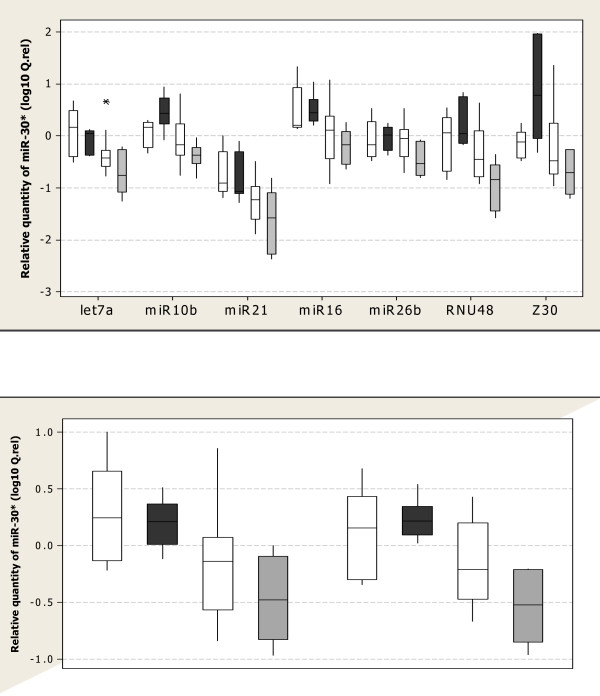
Boxplot of miR-30* relative quantities in benign (BEN, clear), bone metastases (BM, dark), metastases free (MF, dashed) and visceral and bone metastases (VBM, shaded) tissues using different normalisation strategies. (Q. rel = E ^-ΔΔCt^). The boxes represent the interquartile range. The line drawn through the boxes represents the median. Whiskers extend to the highest and lowest values in the data set. **(A) **miR-30* normalised using each EC individually. MiR-30* expression was significantly different between the tissue subgroups (P < 0.05) except when using miR-26b as a single EC. **(B) **miR-30* normalised using geNorm's top two recommended ECs (2 × ECs = let-7a and miR-16) and geNorm's top five recommended ECs (5 × ECs = let-7a, miR-16, miR-26b, miR-21 and RNU48). miR-30* was differentially expressed between groups using either the top 2 or the top 5 most stable ECs (p < 0.05). A significant difference was detected between the MF and VBM groups using the 5 EC gene approach, this was not detected when using the top 2 ECs for normalization.

GeNorm generates a gene-stability measure (M) which may be defined as the average pairwise variation (V) for one candidate EC gene compared to all other candidate EC genes. Stepwise exclusion of the gene with the highest M value results in recalculation of M values for the remaining genes and ultimately, the identification of the most stable pair [30]. The wide range in M values depicts the high variability detected in candidate EC gene stability (Table 2). The differences in miR-30* expression detected between the tissue groups varied greatly depending on which single EC was used for normalisation. For example, in the BEN and BM breast tissues, the expression of miR-30* could be made to appear up- or down-regulated relative to normal breast tissue depending on the EC gene used (Fig. 2A). These results draw particular attention to the potential effect of EC choice on the outcome of a study and demonstrates the need for validation of candidate ECs to produce reliable expression data. In geNorm, a normalisation factor (NF) is generated for each sample using the geometric average of the expression of the most stable EC genes. The pairwise variation value, V is the variation between two sequential NFs (V_n/n+1_, where n = the number of ECs used). The recommended pairwise variation of 0.15 is a guideline value and is not intended as an absolute cut-off. This guideline value may not always be achievable [56] but should be considered, particularly if small expression differences are to be measured. The lowest pairwise variation value was achieved when the top 5 candidate genes were used as ECs (0.220, Fig. 3B). Let-7a and miR-16 were identified as the most stable pair of EC genes using geNorm. Significant differences in miR-30* expression were detected between the tissue groups using either the top two ECs (P = 0.01) or the top five ECs (P = 0.002, Fig. 2B). The *post-hoc *analyses revealed both normalisation approaches detected significant differences between the BM vs MF groups, the BM vs VBM groups and the BEN vs VBM groups but each approach detected an additional intergroup difference not detected by the other approach. The number of genes to use in a normalisation strategy is in most cases, a trade off between required resolution and practicality and for most purposes the EC gene combination let-7a and miR-16 should suffice. Both genes had the lowest stability values, as determined by geNorm and NormFinder (Table 2).

A tumour suppressor role for let-7a in lung tissues seems likely due to its widespread downregulation in tumour versus normal lung tissues as well as the identification of an oncogenic target, RAS, in this tissue [57]. However, it is unclear whether let-7a is implicated in breast cancer since the results of recent studies have been equivocal [57]. Whilst deletion of the *miR-16 *gene has been implicated in the development of chronic lymphocytic leukemia [7], a specific role for this miRNA in breast cancer has not been identified. From a panel of 345 miRNAs, miR-16 was selected in the top 15 most stably-expressed miRNAs across 40 normal human tissue types [58]. A microarray study [51] which looked at the expression of 287 miRNAs in various normal and tumour tissues, not including breast tissue, selected a panel of suitable EC genes based on a number of criteria including high and consistent expression of the miRNA across the tissues. Depending on the tissue sample set, both let-7a and miR-16 were ranked in the top 10–15 most stably-expressed miRNAs, supporting the findings of the present study.

The tissues used in this study are clinically and pathologically diverse (see Table 3) making this study of interest to a broad spectrum of the breast cancer research community. Recent findings would suggest that, unlike mRNAs, the miRNA fraction present in FFPE tissues is relatively unaffected by the fixation process and that miRNAs extracted from these tissues may be accurately profiled using RQ-PCR [27]. Thus, the ECs identified in this study may also prove useful for miRNA RQ-PCR analysis of FFPE breast tissues.

## Conclusion

MiRNA expression studies utilising RQ-PCR should begin with the careful selection of appropriate ECs for normalisation to ensure accurate quantitation of this very exciting class of molecules. This study indicates an appropriate strategy to validate ECs for any miRNA RQ-PCR study and has identified a reliable two-gene normaliser for use in breast cancer studies. We recommend the combined use of Let-7a and miR-16 in this context.

## Methods

### Tissue Cohort

Primary breast tumour tissues (n = 31) were obtained from patients during primary curative resection, at Galway University Hospital, Galway, Ireland. Samples were categorised into benign (n = 5) or malignant groups (n = 26) according to standard histopathological parameters. The malignant breast tumour tissues were divided into three groups depending on the patient's disease progression in the five years following removal of the primary tumour; tumours from patients who did not develop metastases (metastases-free, MF, n = 13), those from patients who developed bone metastases (BM, n = 7) and those from patients who developed visceral and bone metastases (VBM, n = 6).

Clinical data relating to the malignant tumour tissues used in this study are shown in Table 3. RNA from normal tissues (n = 5), recovered from patients undergoing reduction mastopexy surgery were used as calibrator samples for RQ-PCR analysis. Tissues were immediately snap-frozen in liquid nitrogen and stored at -80°C until RNA extraction. Prior written and informed consent was obtained from each patient and the study was approved by the ethics review board of Galway University Hospital. Clinical data were obtained from the Breast Cancer Database at the Department of Surgery, Galway University Hospital.

### RNA Extraction

Approximately 100 mg of tissue was homogenised in 1–2 mL QIAzol (Qiagen, Crawley, UK) using a bench-top homogeniser (Polytron PT1600E, Kinematica AG, Littau-Luzem, Switzerland. Large (> 200 nt) and small RNA (< 200 nt) fractions were isolated separately using the RNeasy^® ^Plus Mini Kit and RNeasy MinElute^® ^Cleanup Kit (Qiagen, West Sussex, UK) according to the Supplementary Protocol: Purification of miRNA from animal cells. A portion of the purified large and small RNA was aliquotted for quantitative and qualitative analysis using NanoDrop1000^® ^spectrophotometry and the Agilent 2100 Bioanalyzer respectively. The remaining RNA was stored at -80°C until further use.

### RNA Analysis

**Figure 4:**
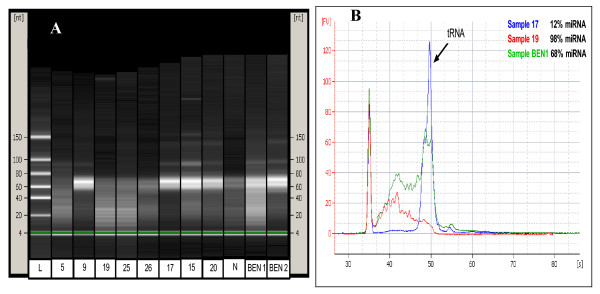
**Results of Agilent Bioanalyser Small RNA assay**. **(A) **Virtual gel-numbered samples refer to malignant breast tissues (as per Table 2), L = Ladder, N = Normal breast tissue, BEN = benign breast tissue. The lower marker is visible at 4 nt. Samples with a large percentage (> 75%) of miRNA (10–35 nt) include the samples BEN1, 19 and 25, the latter two belonging to the bone metastases (BM) patient group. The high intensity band in samples 9, 15, 17, 20, BEN1 and BEN2 between 60 and 80 nt represents high recovery of tRNA (73–93 nt). In general, these samples had a lower percentage of miRNA. **(B) **Electropherogram. Numbered samples refer to malignant breast tissues (as per Table 2), BEN = benign breast tissue.

MicroRNA concentration and purity were assessed using the NanoDrop1000^® ^spectrophotometer (NanoDrop Technologies Inc, Wilmington, DE, USA). The small-RNA enriched fraction was analysed using the Small RNA Assay with the Agilent 2100 Bioanalyzer (Fig. 4) (Agilent Technologies, Palo Alto, CA, USA). For this assay, samples were diluted to 1 ng/μL, within the quantitative and qualitative range of the assay. Integrity of the large RNA fraction (> 200 nt) was assessed using the RNA 6000 Nano LabChip Series II Assay (Agilent Technologies). An RNA integrity number (RIN) is generated for each sample based on the ratio of ribosomal bands and also the presence or absence of degradation products on the electrophoretic image. A threshold value of RIN ≥ 7 was applied.

### Candidate Endogenous Control Genes

The small nuclear and small nucleolar genes were chosen from the ten Human TaqMan MicroRNA Assay Controls available from Applied Biosystems (Foster city, CA, USA) at the time of study. The five miRNA genes were selected based on their known expression in breast cancer tissues and/or based on their previous use as an EC gene in a breast cancer study [24]. Known functions of the candidates are listed in Table 4.

**Table 4:** Details of candidate endogenous control (EC) genes and their PCR amplification efficiencies

**Name**	**Mature** **length** **(nt)**	**RNA** **species**	**Accession** **number**	**Function**	**Reference**	**PCR** **Amplification** **efficiency (%)**
let-7a	22	miRNA	MI0000060*	Negatively regulates RAS oncogene	[57]	96.3
miR-10b	22	miRNA	MI0000267 *	No functionally-verified targets		104.1
miR-16	22	miRNA	MI0000070 *	Negatively regulates B-cell lymphoma mRNA in chronic lymphocytic leukaemia patients	[7]	104.3
miR-21	22	miRNA	MI0000077 *	Antiapoptotic, negatively regulates apoptosis-related genes	[42]	96.8
miR-26b	22	miRNA	MI0000084 *	No functionally-verified targets		98.1
RNU19	198	snoRNA	X94290 **	May be involved in pre-rRNA processing	[59-61]	99.2
RNU48	63	snoRNA	NR_002745 **	Guides the 2'O-ribose methylation of 28S rRNA	[62]	108.9
Z30	97	snoRNA	AJ007733 **	Guides the methylation of the Am47 residue in U6 snRNA	[63]	104.1

*mirBase database accession number ** Entrez gene ID

### cDNA Synthesis and RQ-PCR

Each reaction was primed using a gene-specific stem-loop primer. The primer and probe sequences for let-7a, miR-10b, miR-16, miR-26b and miR-30* were as previously published [18]. Where sequences were available, primers were obtained from MWG Biotech (Ebersberg, Germany). Otherwise, assays containing the RT stem-loop primer and the PCR primers and probes were used (Applied Biosystems, Foster City, CA, USA). Small RNA (5 ng) was transcribed using MultiScribe Reverse Transcriptase (Applied Biosystems). The reaction was performed using a GeneAmp PCR system 9700 thermal cycler (Applied Biosystems). An RT-negative control was included in each batch of reactions. The PCR reactions were carried out in a final volume of 20 μL using an ABI Prism 7000 Sequence Detection System (Applied Biosystems). Briefly, reactions consisted of 1.33 μL cDNA, 1× TaqMan Universal PCR Master mix, No AmpErase UNG, 0.2 μM TaqMan^® ^Probe (Applied Biosystems), 1.5 μM forward primer and 0.7 μM reverse primer. The PCR reactions were initiated with a 10 min incubation at 95°C followed by 40 cycles of 95°C for 15 sec and 60°C for 60 sec. cDNA, synthesised from pooled normal breast tissue, was included on each 96-well plate as an interassay control and calibrator. All reactions were performed in triplicate. The threshold standard deviation for intra- and inter-assay replicates was 0.3. PCR amplification efficiencies were calculated for each candidate EC RQ-PCR assay using the formula E = (10^-1/slope^-1) × 100, using the slope of the plot, Ct versus log input of cDNA. PCR amplification efficiencies for each EC candidate are shown in Table 4.

### Data Analysis

Relative quantities (Q.rel) for each candidate EC gene were calculated from cycle threshold (C_t_) values scaled to a calibrator sample (pool of 5 normal tissues) and corrected for efficiency of amplification (E) according to the formula: Q.rel. = E^-ΔCt^, with ΔCt = average Ct test sample-average Ct calibrator sample. To calculate the expression of the target gene miR-30* relative to each of the EC candidates, the ΔΔCt method was used, with ΔΔCt = (Ct target gene, test sample-Ct endogenous control gene, test sample)-(Ct target gene, calibrator sample-Ct endogenous control gene, calibrator sample). Again, quantities were corrected for efficiency of amplification and the errors were calculated as previously described [30].

Stability of EC expression was analysed using two freely-available programmes; geNorm and NormFinder. GeNorm (vs. 3.4) is a Visual Basic Application for Excel that calculates a gene-stability measure (M) for all candidate EC genes in a given set of samples and determines the most reliable pair of ECs, showing greatest stability of expression ratio across samples. It is based on a pair-wise comparison model. NormFinder [54], an excel-add-in, uses an ANOVA-based model to estimate intra- and inter-group variation. It combines these estimates to produce a stability value for each candidate. NormFinder indicates the single most stable EC and EC pair where the stability of the latter is greater than that of the single EC. Prior to geNorm and NormFinder analysis, Ct values were converted into Q.rel values (E = ^-ΔCt^), as detailed above. For Normfinder analysis samples were grouped into metastases free (MF, n = 13), bone metastases (BM, n = 7), visceral and bone metastases (VBM, n = 6) and benign (BEN, n = 5) as described above. Statistical analyses were performed using Minitab (vs 15; Minitab Ltd., Coventry, UK). Distribution of data was determined using the Anderson-Darling normality test and parametric tests were used where appropriate. Levene's statistic was used to assess if there was a significant difference in variance between genes. The equivalence test was used to assess whether genes were equivalently expressed between tumour (benign and malignant) and normal breast tissues and between malignant and benign breast tissues [41]. ANOVA, Fisher's least significant difference tests and Kruskal Wallis tests were applied to determine the effect of EC on target gene expression. P values < 0.05 were considered statistically significant.

## Authors' contributions

PAD performed the experiments, was responsible for data analyses and drafted the manuscript. REM contributed throughout the experiment, critically reviewed the manuscript and participated in data analysis. AL contributed to RQ-PCR analysis and preliminary data analysis. MJK participated clinically in sample provision and in critical examination of the manuscript. NM conceived, designed and supervised experimental work and manuscript editing. All authors read and approved the final manuscript.
